# Evaluation of Heavy Metal (Lead, Mercury, Cadmium, and Manganese) Levels in Blood, Plasma, and Urine of Adolescents With Aggressive Behavior

**DOI:** 10.7759/cureus.33902

**Published:** 2023-01-17

**Authors:** Süleyman Yıldız, Ayfer Gözü Pirinççioğlu, Enes Arıca

**Affiliations:** 1 Pediatrics and Child Health, Mardin Derik State Hospital, Mardin, TUR; 2 Department of Pediatrics and Adolescents Health, Dicle University, Diyarbakır, TUR; 3 Department of Forensic Medicine, Dicle University, Diyarbakır, TUR

**Keywords:** adolescents’ health, violent, manganese, cadmium, mercury, lead, aggression, intoxication, heavy metal, adolescent

## Abstract

Background

Heavy metals can cause health problems by affecting the biological structure even at very low concentrations. Asymptomatic heavy metal poisoning causes non-specific symptoms such as behavioral disorders, difficulty in learning, and aggressive behaviors. There is also a great concern about the incidence of aggressive behavior among adolescents. A few research studies have concluded that a complex interaction or combination of factors leads to an increased risk of aggressive behavior in adolescents. This study aims to determine the correlation between the heavy metal levels in blood, plasma, and urine and the aggression level in adolescents.

Materials and methods

Two hundred twenty-eight adolescents between the ages of 13 and 19 were enrolled in the study. Blood, plasma, and urine heavy metal levels of the participants were measured by an inductively coupled plasma mass spectrometer (ICP-MS) device (Model 7700x; Agilent, Santa Clara, CA, USA). Buss and Perry’s aggression questionnaire was used to investigate the correlation between heavy metals and aggressive behaviors in adolescents.

Results

Lead blood (r=0.34, p<.01), lead plasma (r=0.22, p<0.01), lead urine (r=0.31, p<.01), mercury blood (r=0.35, p<0.01), mercury urine (r=0.21, p<0.01), manganese blood (r=0.34, p<0.01), manganese plasma (r=0.33, p<0.01) and manganese urine (r=0.39, p<0.01) were positively correlated with tendency to aggression whereas no significant relationship was found between cadmium in blood, plasma, urine and mercury in plasma with aggression.

Conclusion

The study showed valuable data to associate a significant relationship between adolescents' aggression levels and heavy metals. There is an undeniable relationship between the health of adolescents and environmental pollution caused by heavy metals. Therefore, taking the necessary measures to prevent environmental heavy metal pollution is crucial for adolescent health.

## Introduction

The environment is one of the most important factors affecting human health. In 2012, 1.7 million deaths in children under the age of five were attributed to the environment. According to the World Health Organization (WHO), one in four child deaths can be prevented by reducing environmental risks [1].

Heavy metals, one of the important causes of environmental pollution, can cause health problems by affecting the biological structure even at very low concentrations. Children with asymptomatic heavy metal toxicity caused by prolonged low-dose exposure may exhibit nonspecific symptoms such as behavioral disturbances, learning and perception difficulties, and a tendency to violence [2]. Some studies have stated that repeated exposure to heavy metals increases the tendency to crime and violence, especially during adolescence. They have shown that among those with prenatal or postnatal exposure to heavy metals, the frequency of social introversion, depression, neurological problems, aggression, sleep disturbance, and even substance abuse and forensic events is increased [3].

Adolescence is the phase of life between childhood and adulthood, from ages 10 to 19. Approximately 1.2 billion adolescents live in the world, and in some countries, adolescents make up a quarter of the population. This population also constitutes a large group that is ignored and neglected in many health systems. It is known that an estimated 1.1 million adolescents die each year from preventable causes. Adolescence is a period that begins with sexual and psychosocial maturation caused by physical and emotional processes and ends with the individual gaining independence, a sense of identity, and social productivity. During this period, rapid developmental changes occur in biological, psychological, and social fields, and difficulties specific to this period may be experienced. One of the difficulties particular to this period is the tendency to aggressive behaviors [4]. Some risk factors that may increase aggression tendency in adolescents have been identified. Among these risk factors, heavy metal toxicity caused by environmental pollution, the importance of which has recently been understood, is highly effective in promoting violent behavior. Reports have demonstrated that heavy metal levels increase in adolescents with violent behaviors parallel to the prevalence of violent tendencies among adolescents [5]. Due to their high toxicity and widespread use, lead, mercury, cadmium, and manganese are among the heavy metals that are important for public health. Although some studies report the determination of heavy metal levels in children, there are very few studies specific to the adolescent age group [6].

This study aims to determine the relationship between the aggression level of adolescents and heavy metal levels in blood, plasma, and urine.

This article study was previously presented as a meeting abstract at the European Academy of Paediatrics Congress on October 16, 2020.

## Materials and methods

The present study was conducted prospectively between June 2019 and November 2019. Ethics approval was obtained from the Ethics Committee of Dicle University, Faculty of Medicine (TIP.19.025). The parents (mothers and/or fathers) of 228 adolescents who presented to the Adolescent Outpatient Clinic of Dicle University Hospital and who met the inclusion criteria were informed about the purpose of the study and given consent forms, and detailed written consents were obtained from those who accepted to participate in the study. Adolescents with a chronic organic or psychiatric illness, regular drug use, metal prosthesis, or amalgam filling were excluded from the study.

Buss-Perry Aggression Questionnaire

Buss and Perry’s aggression questionnaire was used in this study to investigate the correlation between heavy metals and aggressive behaviors in adolescents. This questionnaire has a minimum value of 29 and a maximum value of 145, which were scored with a 5-Likert scale; 5 for 'strongly agree', 4 for 'agree', 3 for 'neither', 2 for 'disagree', and 1 for 'strongly disagree'. It aims to measure four sub-dimensions of aggression: physical aggression, verbal aggression, anger, and hostility. The physical aggression subscale includes nine questions measuring physical harm to others, verbal aggression measurements include five questions about verbal aggression toward others, anger includes seven questions measuring the emotional aspect of aggression, and the hostility scale includes eight questions assessing items related to cognitive aspects of aggression [7]. This scale was previously translated to Turkish and adapted and validated for 27 items by Madran [8]. The general tendency of the aggression of each adolescent was calculated over the total score obtained from 27 items in the scale. Since the participants rate each statement on a 5-Likert scale, the minimum score of a participant will be 27, while the maximum will be 135. In the original questionnaire, participants were divided into three groups depending on their scores on the aggression scale, where the questionnaire involved 34 items. Scores lower than 59 were assigned as low aggression, those between 59 and 110 as moderate aggression, and those higher than 110 as high aggression. In the current study, the grouping based on 27 items was determined by 46 or below as low, between 47 and 87 as moderate, and 88 and above as high aggression tendency [9].

Validity and reliability analyses of the aggression questionnaire

The translation and adaptation as well as the reliability and validity of the aggression questionnaire were previously reported. The validity of this scale for the current study was also tested using the principal component analysis method with the varimax rotation. The analysis results showed that all items were factored on one component, except 16 and 17 with a load less than 0.20. After removing these two items, new factor and reliability analyses were carried out for the remaining 27 items with a Cronbach's Alpha of 0.95, and a total proportion of variance explained of 56.16%.

Sample collection

The patients were questioned about whether they had received any contrast agent such as gadolinium, iodine, or barium in the last 96 hours since these contrast agents are known to interfere with widely used laboratory techniques in the analysis of trace metals. The participants used disposable towels to dry their arms after washing with soap and water. After wiping the relevant skin of the forearm vein with ethyl alcohol, blood samples were taken into heavy metal-free ethylenediaminetetraacetic acid (EDTA) tubes using disposable vacuum needle tips, followed by centrifuging at 4000 rpm for six minutes. The serum samples were then treated with nitric acid and transferred to demineralized polystyrene tubes. The blood collection was carried out by experienced nurses in an allocated and cleaned room for this procedure. Sterile urine sample flasks were employed to collect urine samples from participants who were asked to avoid the possible contamination of the samples. All collected samples were kept at -20 Celcius before analyses.

Analysis of heavy metal levels

Blood, plasma, and urine heavy metal levels were measured by an inductively coupled plasma mass spectrometer (ICP-MS), which is one of the most sensitive methods in quantitative measurements of heavy metals today [10]. Measurements were carried out at Dicle University Science and Technology Application and Research Center (DÜBTAM). Quantification of metals was carried out with a Model 7700x ICP-MS (Agilent, Santa Clara, CA, USA). We utilized previously proposed operating parameters of the ICP-MS system to determine metals in the biological matrix. Metal levels were given by means of micrograms per liter (µg/L or ppb). Multi-element calibration standard 2A solutions (Agilent) at a concentration of 100 mg/L were used. 2A lead, cadmium, manganese, and mercury solutions (Agilent) were utilized to prepare calibration standards at six different concentrations: 1, 5, 10, 25, 50, and 100 µg/L by taking into consideration the real concentrations in our samples of interest. The instrument repeated the calibration for every 50 samples. All elements were studied in "No gas" mode. ICP-MS Internal Std Mix was purchased from Agilent. As an internal standard, 209 Bismuth was used for lead, cadmium, and mercury while 45Sc was for manganese. Nitric acid (HNO3, 65% v:v) was purchased from Merck (Darmstadt, Germany) and was used to prepare calibration standards. Blood, plasma, and urine samples were exposed to microwave digestion by use of Mars Xpress (CEM, Matthews, NC, USA) according to the previously published procedure. Ultrapure water (Direct-Q8; Merck) with a resistivity of 18MΩ cm was utilized to prepare the solutions for the experimental work. Argon gas with a purity of 99.999% was obtained from a local company (Linde Gaz, Diyarbakır, Turkey).

Data analysis and evaluation techniques

The data analysis was carried out using the SPSS version 22 statistical software package (IBM Corp., Armonk, NY, USA). The data were analyzed using the Chi-squared test and the Mann-Whitney U-test. In the study, the level of statistical significance was set at p=0.05 for all data.

## Results

The numbers of females and males were 127 (55.7%) and 101 (44.3%), with a mean age of 15.3±1.8 years. All the participants (100%) were literate; 1.8% (n=4) were primary school students, 40.4% (n=92) were secondary school students, and 57.9% (n=132) were high school students. A large part of the participants, 96.1% (n=219), had declared full immunization while the rest, 3.9% (n=9), were not sure whether they had incomplete immunization or full immunization. A small number of the subjects, 13.2% (n=30), were smokers while 86.8% (n=198) were not. The rate of participants who responded "yes" to our question about whether there is someone who smokes regularly at home was 31.6% (n=72), while the rate of those who responded "no" was 68.4% (n=156). The demographic characteristics of the participants are given in Table 1.

**Table 1:** The demographic characteristics of adolescents *The values within the parenthesis corresponds to those of fathers

	n	%
Sex		
Female	127	55.7
Male	101	44.3
Age (years)		
13	49	21.5
14	42	18.4
15	41	18
16	32	14
17	28	12.3
18	13	5.7
19	23	10.1
Education (Participants)		
Literate	228	100
Primary school	4	1.8
Secondary school	92	40.4
High school	132	57.9
University	0	0
Education (Parents)*		
Uneducated	34 (7)	14.9 (3.1)
Primary school	117 (14)	51.3 (6.1)
Secondary school	44 (98)	19.3 (43)
High school	30 (80)	13.2 (35.1)
University	3 (29)	1.3 (12.7)
Fully vaccinated	219	96.1
Vaccination (Absent/Unknown)	9	3.9
Smoking (Yes)	30	13.2
Smoking (No)	198	86.8
Household smoking (Yes)	72	31.6
Household smoking (No)	156	68.4

The heavy metal levels in adolescents with low, moderate, and high aggression tendencies are given in Table 2. The results revealed that blood and urine levels of mercury and lead and blood, plasma, and urine levels of manganese are significantly different in terms of aggression scores (Table 2). On the other hand, the heavy metal levels in smokers were higher compared to those in non-smokers. However, a significant difference between smokers (X=0.20, SD=0.37) and non-smokers (X=0.01, SD=0.18) was only observed in cadmium levels in urine (t (228) = -2.36, p<0.05).

**Table 2:** Number (N), mean and standard deviation values for heavy metal levels of participants with low, normal or high aggression tendency

Minimum: 27 Maximum: 135	Heavy Metal	N	Mean (µg/L)	Standard deviation
Low (≤ 46)	Lead (Blood)	43	5.5039	5.16755
	Mercury (Blood)	43	0.7578	1.14803
	Cadmium (Blood)	43	0.2181	0.32729
	Manganese (Blood)	43	10.3255	5.72026
	Lead (Urine)	43	1.5763	1.97334
	Mercury (Urine)	43	0.1932	0.37966
	Cadmium (Urine)	43	0.1060	0.18689
	Manganese (Urine)	43	2.0477	1.69458
	Lead (Plasma)	43	0.2011	0.34751
	Mercury (Plasma)	43	0.2410	0.54385
	Cadmium (Plasma)	43	0.0244	0.02878
	Manganese (Plasma)	43	0.5617	0.24877
Normal (47-87)	Lead (Blood)	146	6.0741	6.98122
	Mercury (Blood)	146	0.6063	0.75398
	Cadmium (Blood)	145	0.2213	0.46417
	Manganese (Blood)	146	11.4171	8.90593
	Lead (Urine)	146	1.3861	1.98942
	Mercury (Urine)	146	0.1249	0.19672
	Cadmium (Urine)	145	0.0970	0.21592
	Manganese (Urine)	146	2.5928	2.03756
	Lead (Plasma)	146	0.1611	0.24429
	Mercury (Plasma)	146	0.1320	0.39589
	Cadmium (Plasma)	145	0.0378	0.08680
	Manganese (Plasma)	145	0.6528	0.38247
High (≥88)	Lead (Blood)	39	14.8011	15.84211
	Mercury (Blood)	39	2.5002	3.89780
	Cadmium (Blood)	39	0.2712	0.35193
	Manganese (Blood)	39	21.4937	16.67773
	Lead (Urine)	39	3.7731	4.29710
	Mercury (Urine)	39	0.3565	0.51238
	Cadmium (Urine)	39	0.1626	0.24285
	Manganese (Urine)	39	5.0225	3.70230
	Lead (Plasma)	39	0.3721	0.34010
	Mercury (Plasma)	39	0.2528	0.39851
	Cadmium (Plasma)	39	0.0361	0.02963
	Manganese (Plasma)	39	1.1334	1.02257

The correlations between the variables of the study were analyzed using Pearson's correlation method (Table 3). The results indicated that the blood (r=0.34, p<0.01), plasma (r=0.22, p<0.01) and urine (r=0.31, p<0.01) levels of lead, the blood (r=0.35, p<0.01) and urine (r=0.21, p<0.01) levels of mercury, and the blood (r=0.34, p<0.01), plasma (r=0.33, p<0.01) and urine (r=0.39, p<0.01) levels of manganese were positively correlated with general aggression tendency while the blood, plasma, and urine levels of cadmium and the plasma level of mercury were not significantly correlated with aggression tendency.

**Table 3:** Correlations between the heavy metal levels in blood, plasma and urine using Pearson's correlation method * P < 0.05 ** P < 0.01

		1	2	3	4	5	6	7	8	9	10	11	12	13
1	Aggressive behavior	1												
2	Lead blood	0.34^**^	1											
3	Lead plasma	0.22^**^	0.61^**^	1										
4	Lead urine	0.31^**^	0.85^**^	0.56^**^	1									
5	Mercury blood	0.35^**^	0.48^**^	0.24 ^**^	0.33^**^	1								
6	Mercury plasma	0.05	0.13	0.06	0.11	0.32^**^	1							
7	Mercury urine	0.21^**^	0.19^**^	0.10	0.09	0.55^**^	0.33^**^	1						
8	Cadmium blood	0.05	0.17^*^	0.11	0.16^*^	0.14^*^	-0.05	0.26 ^**^	1					
9	Cadmium plasma	0.02	0.10	0.05	0.08	0.04	0.12	0.03	0.22^**^	1				
10	Cadmium Urine	0.11	0.16^*^	0.15^*^	0.15^*^	0.08	-0.05	0.22^**^	0.78^**^	0.15^*^	1			
11	Manganese blood	0.34^**^	-0.03	-0.00	-0.02	0.02	-0.07	-0.02	-0.07	-0.08	-0.03	1		
12	Manganese plasma	0.33^**^	0.06	0.05	0.08	0.07	0.01	-0.03	-0.00	-0.04	0.02	0.72^**^	1	
13	Manganese urine	0.39^**^	0.17^*^	0.10	0.13^*^	0.23^**^	0.11	0.13	0.14^*^	0.03	0.12	0.51^**^	0.50^**^	1

## Discussion

Because of their high toxicity and widespread use, lead, mercury, cadmium, and manganese are among the heavy metals that have a specific place in public health. These heavy metals are regarded as systemic toxic substances known to cause multiple organ damage in both acute and late stages. Furthermore, even exposure to low doses of these heavy metals causes neuropsychiatric and neurodevelopmental diseases in children [11].

It has been shown that repeated exposure to heavy metals, particularly manganese and lead, plays a role in developing violent behaviors [12]. Although it is known that exposure to other heavy metals such as arsenic, cadmium, chromium, and mercury leads to neurological deficits, there is no clear correlation demonstrated regarding the tendency for aggression and crime. So the studies about the effect of heavy metals on adolescents' aggressive behavior are very limited [13]. Therefore, the current study poses a standing point in the association between asymptomatic heavy metal intoxication and adolescents' aggressive behaviors.

The current study results pointed out that the adolescents with a high aggression tendency had significantly higher blood and urine levels of lead than those with a low aggression tendency. Childhood lead exposure has been associated with impaired neuronal development, neurochemical damage, and abnormalities in the hippocampus and cerebral cortex [14]. Lead exposure has also been associated with behavioral problems in adolescence and a decrease in intellectual capacity. These behavioral problems include anti-sociality, introversion, and aggression [15]. According to one of the studies, high prenatal and childhood blood lead concentrations were found to be more involved in criminal arrests or violent events in adolescence [5]. As a result of the environmental planning in the USA in the 1970s, the amount of lead started to decrease in certain regions, and a significant decrease was observed in the blood lead levels of children living in those regions compared to the previous years. The fact that children in the relevant region who reached adolescence in the 1990s were significantly less involved in forensic events caused the hypothesis that the individual may exhibit more aggressive behaviors as the level of heavy metal increases in the body [16].

The data of our study indicated that adolescents with a high aggression tendency had significantly higher blood mercury levels than those with a low aggression tendency. Besides, adolescents with a high aggression tendency had significantly higher blood and urine mercury levels than those with a moderate aggression tendency. WHO considers mercury among the top 10 chemicals that pose a significant threat to intrauterine and early child development and pose the greatest threat to human health. All forms of mercury have toxic effects on the nervous, digestive and immune systems as well as the lungs, kidneys, skin, and eyes. Neurodevelopmental and cognitive deficits have been reported in children with prenatal or postnatal low-level mercury exposure [17]. Events in Minamata and Iraq have shown that chronic mercury poisoning can cause significant neurological problems in infants [18]. There was also a significant difference between those who had full immunization (n=219) and those who were incomplete/unknown immunization (n=9) only in terms of plasma mercury. According to the results, adolescents with full immunization had higher plasma mercury levels than those with incomplete or unknown immunization. In recent years it has been suggested, especially by anti-vaccine activists, that thimerosal, which is included in vaccines, is associated with neurodevelopmental diseases. Thimerosal is a compound containing 49.6% ethyl mercury and has been used as an antibacterial for many years. Although many European countries have removed thimerosal from vaccines years before the USA, some studies conducted in these countries have shown that the increase in the prevalence of autism still exhibits an increase similar to that in the world. In other words, the removal of thimerosal from vaccines has not caused any decrease in the incidence of autism [19,20].

Although it is known that exposure to cadmium leads to neurological deficits, there is no clear data on the tendency for aggression and crime [13]. Our results showed that the blood, urine, and plasma levels of cadmium did not have a statistically significant effect on the aggression level of adolescents. When the heavy metal levels of smokers and non-smokers were compared in our study, a significant difference was found only in terms of urine cadmium levels. According to these results, smokers had higher urine levels of cadmium than non-smokers. In this group, only one of the participants had a urine cadmium level (1.60 µg/L) higher than the normal reference value set by the London-based Pathology and Laboratory Medicine (PaLM) international laboratory center [21]. Notably, this adolescent as well as his family members (parents) smokes at home. Because smoking causes significant increases in the concentrations of cadmium in the kidney, which is the main target organ for cadmium toxicity, this adolescent was considered for the follow-up.

Our study demonstrated that adolescents with a high aggression tendency had significantly higher blood, plasma, and urine levels of manganese than those with a low aggression tendency. Besides, adolescents with a high aggression tendency had significantly higher blood and plasma levels of manganese than those with a moderate aggression tendency.

Manganese, which is known to be one of the least toxic metals when taken orally, can accumulate in brain neurons when inhaled. A significant increase has been observed in the fighting and aggressive behaviors of mice exposed to manganese [22]. Anxiety, aggression, emotional instability, psychosis, and fatigue are prominently seen in humans after environmental exposure to manganese. A study conducted in 2007-2009 revealed a positive correlation between the increased levels of manganese measured from the hair of children who were known to be exposed to high amounts of waterborne manganese and their behavioral problems. The biological effects of manganese on behavior are attributed to the accumulation of neurotransmitters. Moreover, increased manganese levels have been found to result in a decreased amount of serotonin and dopamine in the brain, and it is known by studies that decreased amounts of these two hormones in the body increase depression and aggressive behavior [23]. Since changes occur in the release of neurotransmitters such as serotonin and dopamine in the prefrontal region where emotional stimuli are processed during adolescence, this effect of heavy metals such as manganese on neurotransmitters is believed to have much more important consequences. While dopamine receptors increase in teens, serotoninergic receptors decrease. These changes make individuals more emotional, more impulsive, and also less responsive to reward. Decreased sensitivity to reward leads adolescents to take risks and try new things. This has led to the view that changes in neurotransmitters cause some behavioral problems [24].

Although the prospective design of our study is its important strength, it was not possible to analyze all variables related to heavy metals such as diet, physical activity, and internal and external environmental factors. The relatively small number of participants, the absence of different ethnic origins, the limited number of samples, and possible selection bias are the limitations of this study.

## Conclusions

During adolescence, rapid developmental changes occur in the biological, psychological, and social domains, and various challenges specific to this period may be experienced. For example, some risk factors can increase the tendency for aggression. Among these risk factors, heavy metal toxicity caused by environmental pollution, the significance of which has been recently recognized, is quite effective in the development of violent behaviors. Our study demonstrated that the general aggression tendency increases in adolescents as the levels of lead (blood, plasma, urine), mercury (blood, urine), and manganese (blood, plasma, urine) increase in the body. However, no significant correlation was found between cadmium (blood, plasma, urine) or plasma mercury and aggression.

Insidiously progressive heavy metal toxicity can be the underlying cause of many health problems. Heavy metal toxicity can be caused by the food we eat, the cosmetics we use, various plastic and metal mixtures, and even the air we breathe. In other words, we are exposed to heavy metals in all areas of modern life. This poses a risk, especially for children and adolescents, because children and adolescents are more susceptible to chronic heavy metal toxicity than adults and are affected at much lower blood concentrations.

There is an undeniable relationship between the health of children and adolescents and environmental pollution caused by heavy metals. Therefore, taking the necessary measures to prevent heavy metal pollution in the environment is crucial for adolescent health.
